# Biochemical Characterization and Differential Expression of *PAL* Genes Associated With “Translocated” Peach/Plum Graft-Incompatibility

**DOI:** 10.3389/fpls.2021.622578

**Published:** 2021-02-19

**Authors:** Rihab Amri, Carolina Font i Forcada, Rosa Giménez, Ana Pina, María Ángeles Moreno

**Affiliations:** ^1^Department of Pomology, Estación Experimental de Aula Dei - CSIC, Zaragoza, Spain; ^2^IRTA Fruitcentre, PCiTAL, Lleida, Spain; ^3^Unidad de Hortofruticultura, Centro de Investigación y Tecnología Agroalimentaria de Aragón (CITA), Zaragoza, Spain; ^4^Instituto Agroalimentario de Aragón - IA2 (CITA-Universidad de Zaragoza), Zaragoza, Spain

**Keywords:** carbohydrates, enzymatic activities, *PAL* genes, phenolics, phenylpropanoids

## Abstract

Grafting is an ancient plant propagation technique widely used in horticultural crops, particularly in fruit trees. However, the involvement of two different species in grafting may lead to lack of affinity and severe disorders between the graft components, known as graft-incompatibility. This complex agronomic trait is traditionally classified into two categories: “localized” (weak graft unions with breaks in cambial and vascular continuity at the graft interface and absence of visual symptoms in scion leaves and shoots) and “translocated” (degeneration of the sieve tubes and phloem companion cells at the graft interface causing translocation problems in neighboring tissues, and reddening/yellowing of scion leaves). Over the decades, more attention has been given to the different mechanisms underlying the “localized” type of graft-incompatibility; whereas the phenylpropanoid-derived compounds and the differential gene expression associated with the “translocated” graft-incompatibility remain unstudied. Therefore, the aim of this study was to shed light on the biochemical and molecular mechanisms involved in the typical “translocated” graft-incompatibility of peach/plum graft-combinations. In this study, the “Summergrand” (SG) nectarine cultivar was budded on two plum rootstocks: “Adara” and “Damas GF 1869”. “Translocated” symptoms of incompatibility were shown and biochemically characterized in the case of “SG/Damas GF 1869” graft-combination, 3 years after grafting. Non-structural carbohydrates (soluble sugars and starch), phenolic compounds and antioxidant activity, were significantly enhanced in the incompatible graft-combination scion. Similarly, the enzymatic activities of the antioxidant enzyme peroxidase, the phenylalanine ammonia-lyase (PAL) and polyphenol oxidase involved in the phenylpropanoid pathway were significantly affected by the incompatible rootstock “Damas GF 1869”, inducing higher activities in the scion than those induced by the compatible rootstock “Adara”. In addition, a positive and strong correlation was obtained between total phenol content, antioxidant capacity and the expression of the key genes involved in the phenylpropanoid pathway, *PAL1* and *PAL2*. Regarding the “SG/Adara” graft-combination, there were neither external symptoms of “translocated” incompatibility nor significant differences in the biochemical and molecular parameters between scion and rootstock, proving it to be a compatible combination. The differential expression of *PAL* genes together with the biochemical factors cited above could be good markers for the “translocated” peach/plum graft-incompatibility.

## Introduction

Grafting is the association of two genetically different parts: a scion and a rootstock, living in symbiosis to produce a plant with desirable characteristics of both genotypes (Hartmann et al., 2011; Pina et al., 2017). The survival of a grafted plant depends primarily on the success of the graft-union establishment to enable the formation of callus tissue and the vascular connections between the rootstock and the scion (Baron et al., 2019). However, the range of application of grafting is restricted by anatomical, physiological and biochemical factors leading to graft-incompatibility (Zarrouk et al., 2010; Pina et al., 2017).

Apart from the total incompatibility characterized by a complete failure of grafting, Mosse (1962) described two different types of graft-incompatibility: “translocated” and “localized”. The first type is usually expressed during the first year after grafting as growth cessation and leaf chlorosis leading to leaf yellowing/reddening and early defoliation in the budded scion and a radicular system not fully developed (Moreno et al., 1993; Zarrouk et al., 2006; Hartmann et al., 2011; Neves et al., 2017). In peach/plum combinations, this form of incompatibility has been associated with both biochemical and functional alterations at the graft interface, inducing a carbohydrate blockage at the scion, above the graft union (Moing and Carde, 1988; Moing et al., 1990). Nevertheless, these incompatibility symptoms can occur at a later stage of development (Moreno et al., 1993) and some peach/plum combination can exhibit “localized” incompatibility (Salesses and Bonnet, 1992; Zarrouk et al., 2006). The second type is characterized by anatomical irregularities at the graft-union interface accompanied by poor vascular connections leading to mechanical weakness and subsequent breakdown of the graft-union that can occur at a later stage of development (Reig et al., 2019). Some peach/plum graft combinations showed the coexistence of both incompatibilities and the “translocated” incompatibility preceded the occurrence of the “localized” incompatibility (Zarrouk et al., 2006).

In spite of the increasing interest of using new rootstocks in most horticultural crops (Melnyk and Meyerowitz, 2015), biochemical and molecular factors underlying graft-incompatibility are still not well-understood (Pina et al., 2017). The phenylpropanoid-derived compounds play a major biochemical role in plant resistance toward various stresses (Dixon et al., 2002). Nevertheless, the mechanism of their synthesis is still not fully understood, especially regarding the expression and regulation of key genes (Zhang et al., 2016). In plants, the tolerance to biotic and abiotic stresses is usually regulated by phenolic compounds, which are the main secondary metabolites synthesized by the phenylpropanoid pathway (Lattanzio, 2013). Polyphenol oxidase (PPO) and phenylalanine ammonia-lyase (PAL) are two important enzymes involved in the phenylpropanoid pathway and related with the phenolic compounds metabolism. These enzymes, together with the antioxidant enzyme peroxidase (POX), are developmentally and tissue-specifically regulated and may be induced by graft-incompatibility (Zarrouk et al., 2010; Xu et al., 2015). Previously, the content of phenolic compounds has been associated, in general, with the “localized” type of graft-incompatibility, limiting the proliferation and differentiation of callus and the formation of the new vascular tissues in pear (Machado et al., 2017), cherry (Güçlü and Koyuncu, 2012) and apricot (Pereira et al., 2018). In addition, those compounds escape from the vacuole into the cytoplasm where are susceptible to oxidation by PPO and POX enzymes (Feucht and Treutter, 1989), resulting in the production of quinones and polymeric melanins that may polymerize to toxic compounds (Poëssel et al., 1980).

Recently, more attention has been focused to the molecular mechanisms involved in generating a different response between compatible and incompatible graft-combinations (Chen et al., 2017; Ren et al., 2018). Several studies have reported that differential expression of phenylalanine ammonia lyase (*PAL*) genes implies distinct roles in development of graft incompatibility symptoms in different species and could be good markers for the study of compatibility due to its importance in the biosynthesis of many phenolic compounds (Pereira et al., 2014; Irisarri et al., 2016). Other recent reports investigated the transcriptomic profiles during graft union development (Cookson et al., 2013; Mo et al., 2018) and incompatibility responses (He et al., 2018; Assunção et al., 2019) in different woody species. They identified transcripts differentially expressed at the graft interface involved in hormone signal transduction, cell proliferation and elongation, xylem differentiation and other metabolisms, which have advanced our knowledge of graft union formation and graft compatibility.

In the last decades, there has been substantial progress in selecting new plum based rootstocks (mainly *P. cerasifera* Ehrh. and *P. insititia* L.) in breeding programs for different stone fruit species in Mediterranean temperate areas (Byrne et al., 2012; Reig et al., 2018a; Guajardo et al., 2020). The interest in plum species as rootstocks for peach and nectarine cultivars is due to their tolerance to waterlogging, iron-induced chlorosis and salinity, their resistance to root-knot nematodes and their ability to overcome replanting problems comparing with peach seedlings and almond × peach hybrid rootstocks (Jiménez et al., 2011; Mestre et al., 2015; Font i Forcada et al., 2020).

Although phenolic compounds and genes related to the “localized” graft-incompatibility have already been studied in different *Prunus* species (Pina and Errea, 2008; Irisarri et al., 2015), to the best of our knowledge, the mechanism of the phenylpropanoid-derived compounds and the differential gene expression associated with the “translocated” peach/plum graft-incompatibility remain unstudied. Thus, the aim of this study was to assess the biochemical and molecular mechanisms involved in the “translocated” graft-incompatibility of peach budded on plum rootstocks. To fulfill this objective, the analyses of non-structural carbohydrates (soluble sugars and starch), phenolic/antioxidant compounds, enzymatic activities of PAL, PPO and POX and the differential expression of phenylalanine ammonia-lyase genes (*PAL1* and *PAL2*) in response to compatible and incompatible graft-combinations were performed.

## Materials and Methods

### Plant Material and Sampling

The present study was performed in the “Summergrand” nectarine cultivar [*P. persica* (L.) Batsch] budded on two plum rootstocks: “Adara” (“SG/Adara”) and “Damas GF 1869” (“SG/Damas GF 1869”) grown in nursery conditions. “Adara” was selected as a polyvalent rootstock for different stone fruit species (Moreno et al., 1995) from an open-pollinated population of *P. cerasifera* (Myrobalan or cherry plums, 2n = 16). “Damas GF 1869” is a pentaploid hybrid plum rootstock (*P. domestica × P. spinosa*) showing graft-incompatibility with most nectarine and a few peach varieties (Salesses and Al-Kaï, 1985). The two ungrafted plum rootstocks were used as controls. One year old rooted plants of both rootstocks were established in February 2014 at nursery conditions of the Experimental Station of Aula Dei-CSIC (Zaragoza). The “Summergrand” cultivar was T-budded *in situ* in the summer of 2014 on the two rootstocks with 10 replicates (individual trees) for each graft-combination.

Bark tissue samples were collected at leaf fall period (October 2017) and during the most active and strong vegetative period (June 2018) from the stem of each individual tree (*n* = 3) at ± 5 cm approximately above (scion) and below (rootstock) the graft-union for each graft-combination. The samples were frozen immediately in liquid N_2_, lyophilized, ground, and stored at −20°C until further analysis.

### Tree Growth and Leaf Characteristics

The symptoms of “translocated” graft-incompatibility were determined by visual diagnosis of leaves and shoots according to Moreno et al. (1993). In addition, tree growth parameters were determined by tree height and trunk diameter measurements (±5 cm above and below the graft-union) 3 years after grafting. Trunk cross section area (TCSA) was calculated as previously reported by Mestre et al. (2015). The relative content of chlorophyll per leaf area was also estimated in the field using a SPAD-502 Plus chlorophyll meter (Minolta Co., Osaka, Japan) (Süß et al., 2015). Ten representative leaves were randomly selected from the middle of shoots located all around the crown, and SPAD measurements were carried out in three trees per graft-combination according to Zarrouk et al. (2006), at the end of June of the third year after budding.

### Non-structural Carbohydrates (NSC) Analyses

Ten milligrams of the above-mentioned lyophilized bark tissue samples were weighted, mixed with 1 mL of methanol (80%) and kept overnight. The mixture was centrifuged at 20,000 g for 30 min at 4°C (Biofuge Primo R, Heraeus). The supernatant was collected and kept as a stock solution at −20°C. Two hundred microliters of each stock solution were vacuum concentrated in a SpeedVac (Thermo Savant SPD111V) and dry extracts were re-suspended to 200 μL of Milli-Q water. Then, concentrations of the main soluble sugars were determined by high-performance liquid chromatography HPLC (Aminex HPX-87C column, 300 mm × 7.8 mm; Bio-Rad, Barcelona, Spain) with a refractive index detector at 35°C (Waters 2410, Waters Corporation, Milford, MA, United States) as previously reported (Font i Forcada et al., 2013, 2019). Filtered and degassed Milli-Q water was used as mobile phase with a flow rate of 0.6 mL/min at 85°C. Concentrations of the main sugars (fructose, glucose, raffinose, sorbitol, stachyose, sucrose, and xylose) were calculated for each sample. Sugar quantification was carried out with Empower Login software from Waters, using commercial standards (Panreac Química S.A. Barcelona, Spain). The concentration of each individual sugar was expressed as mg per g of dry weight (DW).

The starch extraction was performed according to McGrance et al. (1998) and Zapata et al. (2004), with some modifications. Ten milligrams of the lyophilized samples were mixed with 1 mL of dimethyl sulfoxide DMSO (90%) at 85°C for 1 h. After centrifugation for 15 min at 12,000 g (Heareus Pico 17, Thermo Fisher Scientific, MA, United States), a water dilution 1:10 was prepared and mixed with acidic iodine solution (0.6% KI and 0.03% I_2_ in 0.05 N HCl). After 15 min, the starch content was determined spectrophotometrically at 600 nm. The results were expressed as mg starch per g of DW.

### Phenolic Compounds and Antioxidant Capacity Analyses

From the previously described methanol stock solution, a water dilution 1:10 was prepared and stored at −20°C until further analysis. The analysis of total phenolic content (TPC), flavonoids, anthocyanins and the relative antioxidant capacity (RAC) were carried out using a spectrophotometer Asys UVM 340 Microplate Reader (Biochrom Ltd., Cambridge, United Kingdom) as previously reported (Reig et al., 2016; Font i Forcada et al., 2019).

Total phenolics content (TPC) was determined using the Folin-Ciocalteau reagent according to the method of Singleton and Rossi (1965) with some modifications. Absorbance was measured at 725 nm and the results were expressed as μg of gallic acid (3,4,5-trihydroxy-benzoic acid) equivalents (GAE) per g FW. Flavonoids content was measured employing the colorimetric assay described by Zhishen et al. (1999). Absorbance was measured at 510 nm and results were expressed as catechin equivalents (μg of CE/g DW). Anthocyanins content was determined according to Fuleki and Francis (1968) with some modifications. Aliquots of the extract were acidified with HCl and used for spectrophotometric readings at 535 nm and 700 nm. The results were expressed as μg of cyanidin-3-glucoside equivalents (C3GE) per g of DW using a molar extinction coefficient of ε = 25,965 L/cm mol. The relative antioxidant capacity (RAC) was evaluated using the 2, 2-diphenyl-1-picrylhydrazyl (DPPH) radical scavenging assay according to Brand-Williams et al. (1995). Absorbance was measured at 515 nm, and results were expressed in μg of Trolox equivalents of antioxidant capacity (TEAC) per g of DW.

### PAL, PPO, and POX Enzymatic Extraction and Analysis

Three biological replications of the lyophilized samples (10 mg) were mixed with 10 mL of sodium phosphate buffer (0.1 M, pH 7), containing 1% of polyvinylpyrrolidone (PVP) and 0.5% of Triton X-100. Phenol absorbents (PVP) and detergents such as Triton X-100 were used in the enzymes extraction to prevent the reaction between enzymes and phenolic compounds during extraction (Galeazzi et al., 1981, with some modifications). Samples were centrifuged at 4°C for 15 min at 20,000 g. The supernatant was collected and used as an enzyme extract.

The Phenylalanine ammonia-lyase (PAL) activity was determined by the conversion of L-phenylalanine to trans-cinnamic acid according to the protocol of Tovar et al. (2002) with some modifications. The reaction mixture contained 1 mL of L-phenylalanine solution, 4 mL of sodium phosphate buffer (0.1 M, pH 7) and 1 mL of enzyme extract. After incubated at 40°C for 1 h, the reaction was stopped using trichloroacetic acid (TCA). The cinnamic acid yield was estimated by measuring the absorbance at 290 nm (ε = 17,400 L/cm mol). PAL enzymatic activity was expressed in μmol *trans*-cinnamic acid liberated per g of dry weight (μmol *trans*-cinnamic acid/h g DW).

The polyphenol oxidase (PPO) activity was assayed spectrophotometrically based on the oxidation of catechol to 1.2-benzoquinone (Galeazzi et al., 1981). The reaction mixture consisted of 100 μL of the enzyme extract mixed with 900 μL of catechol dissolved in sodium phosphate buffer (0.1 M, pH 7). The absorbance was measured at 420 nm at different times. PPO activity was calculated as the slope in the linear part of the activity curve obtained. One unit (U) of the enzymatic activity was defined as an absorbance increase of 0.1 per minute under the assay conditions.

The peroxidase (POX) activity was determined by the oxidation of guaiacol (C_3_H_8_O_2_) in the presence of hydrogen peroxide (H_2_O_2_) (Dann and Deverall, 2000). For the development of the reaction, 100 μL of the enzyme extract were mixed with 900 μL of the substrate solution, including guaiacol, H_2_O_2_, and sodium phosphate buffer (0.1 M, pH 7). The reaction rate of POX was estimated with an initial rate of increase in absorbance at 470 nm. One unit of the enzymatic activity (U) was defined as an absorbance increase of 0.1 per minute under the assay conditions.

### Real Time-q PCR Expression Analysis of *PAL* Genes

Total RNA was isolated from 25 mg of the previously frozen lyophilized samples and using the CTAB protocol described by Meisel et al. (2005) with some modifications. The final RNA was re-suspended with DEPC water and stored at −80°C until further analysis. The quality of the total RNA was performed by 1% (w/v) agarose gel using 0.01% SYBR^®^ Safe DNA gel stain (Invitrogen, Thermo Fisher Scientific, United States) and the quantification was determined by an ultraviolet (UV) spectrophotometer (NanoDrop ND-2000, Thermo Fisher Scientific, Wilmington, DE, United States). Prior to cDNA synthesis, the endonuclease DNasa I RNasa-free (ref: EN0521) was used to digest strand DNA, according to the manufacturer’s instructions (Thermo Scientific, Baltics UAB, Lithuania). The total RNA (1 μg) was reverse transcribed using an oligo (dT) 20 and SuperScript^®^ III First Strand cDNA synthesis kit in accordance with the manufacturer’s instructions (Thermo Scientific, Baltics UAB, Lithuania). Real-Time qPCR amplifications were performed on a 7,500 Fast Real Time PCR System (Applied Biosystems, United States, Version 2.0.1) using specific primers for *PAL1* (ppa002099m) and *PAL2* (ppa002328m) as described by Pereira et al. (2014). The reactions comprised a total volume of 20, 10 μL of the SYBR^®^ Green PCR master mix (Applied Biosystems), 10 ng of cDNA and 300 nM of primers. Three technical replications for each of the three biological replicates were analyzed. Four candidate reference genes: *Actin* (*ACT11*), *translation elongation factor* (*TEF2*), *RNA polymerase II* (*RP II*), and *EST* (Gene Bank accession No. DY652828) were tested to identify the most stable reference gene for the normalization of gene expression using a NormFinder algorithm (Andersen et al., 2004). The PCR reactions were identical for all primers sets: 95°C for 10 min followed by 40 cycles at 95°C for 30 s; annealing temperature at 60°C for 1 min, extension at 72°C for 1 min; and a final extension at 72°C for 10 min. Afterward, a dissociation curve was performed with gradient according to the following curve program: 95°C for 15 s, 60°C for 15 s and 95°C for 15 s. The specificity of amplicons was verified by 1% (w/v) agarose gel using 0.01% SYBR Safe DNA gel stain. The efficiencies (E) and the quantification cycles (Cq) values were determined at constant fluorescence using the LingRegPCR software. The relative expression ratio (R) of the two target genes *PAL1* and *PAL2* was calculated based on the E and Cq deviation of each sample vs. a basis sample called control or calibrator, and expressed in comparison to a reference gene as described by Pfaffl (2001).

### Statistical Analysis

Means from three biological replicates for each graft-combination and ungrafted rootstock were analyzed statistically using IBM SPSS 24.0 (United States) software. Data were evaluated by the analysis of variance (ANOVA). When the F test was significant, the separation of means was performed using Duncan’s multiple range test (*P* ≤ 0.05). Correlations between biochemical and molecular parameters, to reveal possible associations, were determined by the average of the three biological replicates, using the Pearson correlation coefficient at *P* ≤ 0.05. Pearson’s rank correlation matrix (*P* ≤ 0.05) was done using the R corrplot package. Principal components analysis (PCA) was performed and a biplot PCA was designed with combined data from both sampling periods using the SPSS software.

## Results

### The Influence of Grafting on Vegetative Growth

**FIGURE 1:**
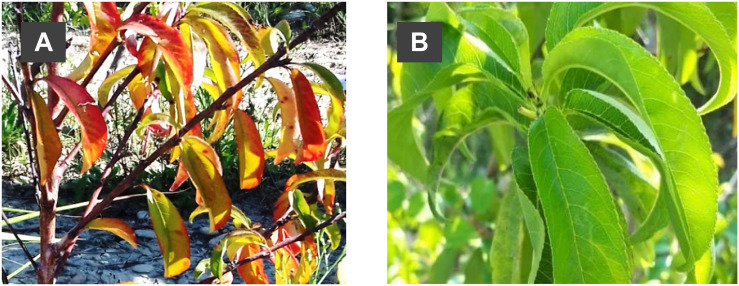
Visual diagnosis of both graft-combinations at the third year after grafting: **(A)** External symptoms of “translocated” incompatibility (curling, reddening and yellowing of leaves) in “SG/Damas GF 1869” graft-combination; **(B)** absence of graft-incompatibility symptoms in “SG/Adara” graft-combination (healthy leaves).

The visual diagnosis of “Summergrand” trees determined that “SG/Adara” exhibited good graft-compatibility, indicated by normal tree height, healthy shoots and leaves, and external bark appearance at the graft-union, until the end of the experiment, 4 years after budding. In contrast, “SG/Damas GF 1869” trees showed typical symptoms of the “translocated” incompatibility, particularly curling, reddening/yellowing of the leaves, unhealthy appearance of shoots, and reduction in vigor (TCSA and tree height) (Table 1 and Figure 1). These symptoms increased progressively from the first year after budding, and became more acute at the end of the experiment (data not shown). In the most severe cases, death of some shoots was observed. Similarly, lower SPAD index values (Table 1) were found in “SG/Damas GF 1869” compared to the compatible graft-combination and the two ungrafted rootstocks.

**TABLE 1:** SPAD values and tree growth parameters determination of “Summergrand” nectarine cultivar grafted on two plum-based rootstocks (“Adara” and “Damas GF 1869”) 3 years after grafting.

	SPAD values	Height (cm)	Scion TCSA (cm^2^)	Rootstock TCSA (cm^2^)
“SG/Adara”	34.0 ± 0.49 b	180.7 ± 11.01 b	14.4 ± 3.70 b	16.6 ± 1.52 b
“SG/Damas GF 1869”	20.9 ± 2.04 a	72.7 ± 2.08 a	2.2 ± 0.97 a	1.9 ± 0.95 a
”Adara” (ungrafted)	34.9 ± 3.31 b	155.7 ± 31.56 b	–	–
”Damas GF 1869” (ungrafted)	37.4 ± 1.32 b	153.7 ± 16.50 b	–	–

*x ± s = mean value ± SD of n = 3 replicates; SD, Standard Deviation.^abc^Mean values in the same column with different letters are significantly different at the P ≤ 0.05 level according to Duncan’s Multiple Range Test.*

### Non-structural Carbohydrates Regulated by Scion-Rootstock Interactions

**FIGURE 2:**
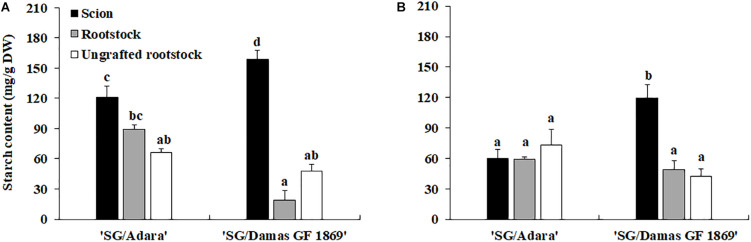
Starch content (mg/g DW) in the scion and rootstock of the compatible (“SG/Adara”) and incompatible (“SG/Damas GF 1869”) graft-combinations, and ungrafted rootstocks [**(A)** October 2017; **(B)** June 2018] 3 years after grafting. Vertical bars represent the mean ± Standard Error (SE) (*n* = 3 replicates). For each graph, ^*abc*^Mean values are significantly different at the *P* ≤ 0.05 level according to Duncan’s Multiple Range Test.

Sugars profile analysis showed that sorbitol and sucrose were the individual soluble sugars identified with higher concentrations in the bark stem of scion and rootstock for both graft-combinations and periods evaluated (Tables 2A,B). Other sugars detected with lower concentrations were glucose and fructose, followed, in general, by stachyose, raffinose and xylose. In the case of the compatible graft-combination (“SG/Adara”), the majority of individual sugars and starch content did not differ significantly in the scion and rootstock except for glucose and fructose (Tables 2A,B). In contrast, the incompatible rootstock “Damas GF 1869” induced a statistically significant increase of starch in the scion for both sampling dates (Figure 2), approximately two-fold higher than the scion on the compatible rootstock in the vegetative period (Figure 2B). In turn, lower sucrose, fructose and sorbitol were shown in the rootstock of the incompatible graft-combination. No significant differences were found in most soluble sugars and starch between the two ungrafted rootstocks. However, they showed the tendency to exhibit higher sucrose and sorbitol but lower fructose and glucose content that both graft-combinations, especially at the vegetative period.

**TABLE 2A:** Stem soluble sugars (mg/g DW) in the bark from the scion and rootstock of the compatible (“SG/Adara”) and incompatible (“SG/Damas GF 1869”) graft-combinations, and ungrafted rootstocks at the leaf fall period.

	Soluble sugar (mg/g DW)							
	Stachyose	Raffinose	Sucrose	Glucose	Xylose	Fructose	Sorbitol	Total sugars
**“SG/Adara”**								
Scion stem	1.76 ± 0.44 a	2.25 ± 0.31 a	22.32 ± 4.04 a	5.30 ± 1.64 b	1.29 ± 0.38 a	7.63 ± 1.74 cd	24.13 ± 4.71 ab	64.68 ± 11.18 ab
Rootstock stem	2.56 ± 1.48 a	2.03 ± 0.25 a	18.53 ± 2.78 a	3.23 ± 0.75 a	0.97 ± 0.08 a	3.87 ± 0.97 ab	21.73 ± 2.09 a	52.92 ± 4.85 a
**“SG/Damas GF 1869”**								
Scion stem	2.74 ± 1.14 a	2.17 ± 1.03 a	20.46 ± 1.63 a	5.38 ± 1.69 b	1.71 ± 1.12 a	9.27 ± 2.38 d	34.32 ± 7.47 bc	76.07 ± 14.05 ab
Rootstock stem	3.79 ± 1.73 a	3.41 ± 1.29 a	17.30 ± 0.70 a	7.37 ± 0.20 c	8.00 ± 2.59 b	6.29 ± 1.76 bc	20.93 ± 1.16 a	67.09 ± 5.90 ab
**Ungrafted rootstocks**								
“Adara”	2.20 ± 0.37 a	0.98 ± 0.15 a	15.97 ± 0.41 a	2.99 ± 0.28 a	0.74 ± 0.12 a	1.75 ± 0.11 a	29.98 ± 3.32 ab	54.62 ± 4.15 a
“Damas GF 1869”	2.07 ± 3.58 a	7.65 ± 3.22 b	24.52 ± 11.7 a	3.07 ± 0.78 a	7.27 ± 4.34 b	2.22 ± 0.46 a	42.47 ± 12.51 c	89.26 ± 29.01 b

*x ± s = mean concentration ± SD of n = 3 replicates.DW, Dry Weight; SD, Standard Deviation.^abc^Mean values in the same column with different letters are significantly different at the P ≤ 0.05 level according to Duncan’s Multiple Range Test.*

**TABLE 2B:** Stem soluble sugars (mg/g DW) in the bark from the scion and rootstock of the compatible (“SG/Adara”) and incompatible (“SG/Damas GF 1869”) graft-combinations, and ungrafted rootstocks at the vegetative period.

	Soluble sugar (mg/g DW)							
	Stachyose	Raffinose	Sucrose	Glucose	Xylose	Fructose	Sorbitol	Total sugars
**“SG/Adara”**								
Scion stem	1.11 ± 1.12 a	3.42 ± 3.26 a	16.54 ± 1.61 ab	10.51 ± 2.47 b	2.14 ± 0.49 a	20.21 ± 3.5 e	25.48 ± 4.12 b	79.42 ± 5.64 bc
Rootstock stem	4.22 ± 1.66 ab	3.70 ± 2.02 a	14.02 ± 4.09 ab	6.97 ± 0.66 ab	1.48 ± 0.27 a	8.48 ± 0.27 bc	17.49 ± 4.04 ab	56.36 ± 10.59 a
**“SG/Damas GF 1869”**								
Scion stem	0.95 ± 0.82 a	1.79 ± 1.27 a	19.79 ± 2.03 bc	5.04 ± 0.57 a	1.56 ± 0.50 a	14.19 ± 1.65 d	26.27 ± 6.13 b	69.59 ± 8.58 ab
Rootstock stem	3.24 ± 3.04 ab	1.94 ± 0.54 a	11.61 ± 2.24 a	8.86 ± 3.38 b	3.15 ± 3.93 a	9.69 ± 4.07 c	14.59 ± 3.59 a	53.08 ± 18.54 a
**Ungrafted rootstocks**								
“Adara”	7.27 ± 1.99 bc	2.29 ± 1.98 a	33.18 ± 7.33 c	5.09 ± 1.94 a	1.44 ± 0.13 a	4.57 ± 1.32 ab	39.68 ± 3.34 c	93.53 ± 8.46 c
“Damas GF 1869”	8.52 ± 1.02 c	4.51 ± 1.16 a	21.78 ± 4.85 bc	5.25 ± 0.42 a	3.25 ± 0.42 a	4.02 ± 0.43 a	36.28 ± 6.47 c	83.6 ± 10.57 bc

*x ± s = mean concentration ± SD of n = 3 replicates.DW, Dry Weight; SD, Standard Deviation.^abc^Mean values in the same column with different letters are significantly different at the P ≤ 0.05 level according to Duncan’s Multiple Range Test.*

### Phenolic Compounds and Antioxidant Capacity

The phenolic compounds content (TPC and flavonoids) and relative antioxidant capacity (RAC) were significantly affected by the “translocated” peach/plum graft-incompatibility. While no significant differences were shown between scion and rootstock in the compatible graft-combination (Table 3), the incompatible rootstock “Damas GF 1869” increased significantly the TPC, flavonoids and RAC activity in the scion. A more acute accumulation of these compounds in the scion was found the following year in late spring (June), with approximately two-fold higher TPC, flavonoids and RAC values than in the rootstock. Regarding the anthocyanin content, there was not a significant effect of the incompatible rootstock on the scion in both sampling dates. No significant differences were found between the two ungrafted rootstocks for all those compounds. However, they showed the tendency to exhibit lower TPC, flavonoids, and RAC values that both graft-combinations, especially at the vegetative period.

**TABLE 3:** Total phenolic content (TPC), flavonoids, anthocyanins, and relative antioxidant capacity (RAC) in the bark from the scion and rootstock of the compatible (“SG/Adara”) and incompatible (“SG/Damas GF 1869”) graft-combinations, and ungrafted rootstocks at leaf fall (October) and vegetative (June) periods.

	TPC (μg GAE/g DW)		Flavonoids (μg CE/g of DW)		RAC (μg TEAC/g DW)		Anthocyanins (μg C3GE/g DW)	
	October	June	October	June	October	June	October	June
**“SG/Adara”**								
Scion stem	11.49 ± 1.42 b	18.05 ± 2.21 b	10.19 ± 1.86 a	17.26 ± 3.71 c	184.7 ± 21.5 b	183.8 ± 7.6 bc	0.29 ± 0.07 ab	0.24 ± 0.18 a
Rootstock stem	12.43 ± 0.80 b	18.32 ± 0.75 b	12.81 ± 1.84 ab	15.79 ± 1.78 bc	174.8 ± 9.8 b	182.2 ± 12.8 bc	0.55 ± 0.14 c	0.23 ± 0.14 a
**“SG/Damas GF 1869”**								
Scion stem	15.72 ± 1.91 c	19.29 ± 0.93 b	13.60 ± 1.04 b	15.17 ± 0.36 bc	238.3 ± 11.6 c	210.9 ± 9.1 c	0.24 ± 0.03 ab	0.11 ± 0.09 a
Rootstock stem	11.30 ± 1.15 b	10.06 ± 1.57 a	11.56 ± 1.19 ab	8.61 ± 1.52 a	174.4 ± 17.2 b	107.5 ± 12.4 a	0.35 ± 0.05 b	0.62 ± 0.17 a
**Ungrafted rootstocks**								
“Adara”	8.85 ± 0.91 a	12.49 ± 2.21 a	11.25 ± 1.34 ab	10.35 ± 2.84 ab	126.1 ± 16.4 a	132.3 ± 28.4 ab	0.29 ± 0.05 ab	0.32 ± 0.16 a
“Damas GF 1869”	8.28 ± 1.69 a	11.73 ± 2.91 a	10.33 ± 3.63 a	9.13 ± 2.52 a	105.1 ± 27.4 a	134.3 ± 37.5 ab	0.18 ± 0.14 a	0.33 ± 0.08 a

*x ± s = mean concentration ± SD of n = 3 replicates.^abc^Mean values in the same column with different letters are significantly different at the P ≤ 0.05 level according to Duncan’s Multiple Range Test.GAE, Gallic Acid Equivalents; CE, Catechin Equivalents; TEAC, Trolox equivalents of antioxidant capacity; C3GE, Cyanidin-3-Glucoside Equivalents; DW, Dry Weight; SD, Standard Deviation.*

### Phenylpropanoid (PAL and PPO) and Antioxidant (POX) Enzymes Activities

**FIGURE 3:**
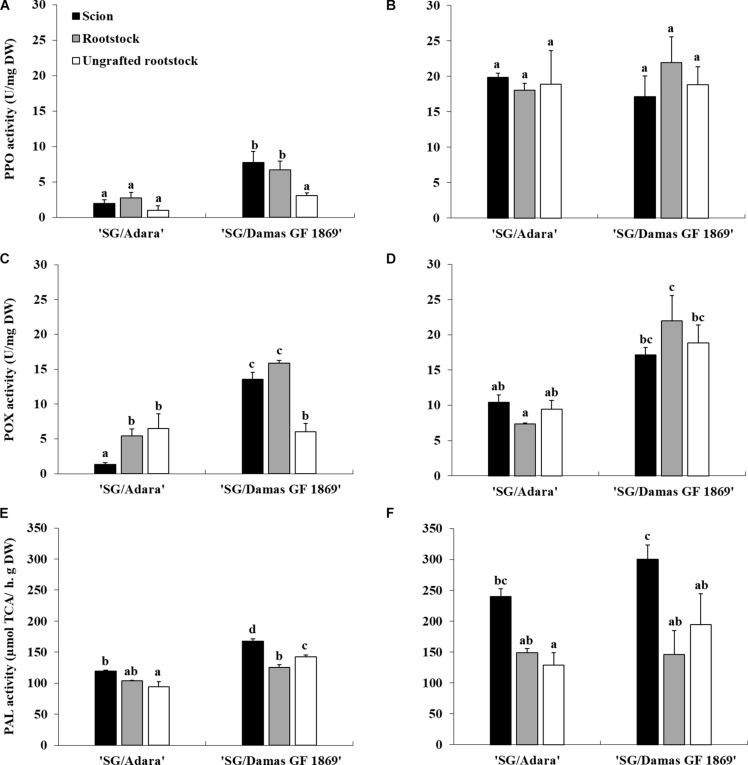
Enzymatic activities of PPO, POX and PAL in the scion and rootstock of the compatible (“SG/Adara”) and incompatible (“SG/Damas GF 1869”) graft combinations, and ungrafted rootstocks [**(A,C,E)** October 2017; **(B,D,F)** June 2018]. Vertical bars represent the mean ± SE (*n* = 3 replicates) 3 years after grafting. For each graph, ^*abc*^Mean values are significantly different at the *P* ≤ 0.05 level according to Duncan’s Multiple Range Test.

Enzymatic assays showed that PAL, PPO, and POX activities were significantly affected by the incidence of the “translocated” graft-incompatibility. While no significant differences were shown, in general, between scion and rootstock in the compatible graft-combination, the incompatible rootstock “Damas GF 1869” increased significantly the PPO, POX, and PAL activity in the scion at leaf fall (Figure 3). Moreover, the incompatible rootstock induced two-fold higher PAL activity in the scion at the vegetative period (Figure 3F). No significant differences were observed between the two ungrafted rootstocks, with the exception for the PAL activity at leaf fall. However, the incompatible rootstock induced approximately four-fold higher PPO and 10-fold higher POX activities in the grafted scion compared to the values of the compatible graft-combination at leaf fall (Figures 3A,C).

### Differential Gene Expression of *PAL* Genes

**FIGURE 4:**
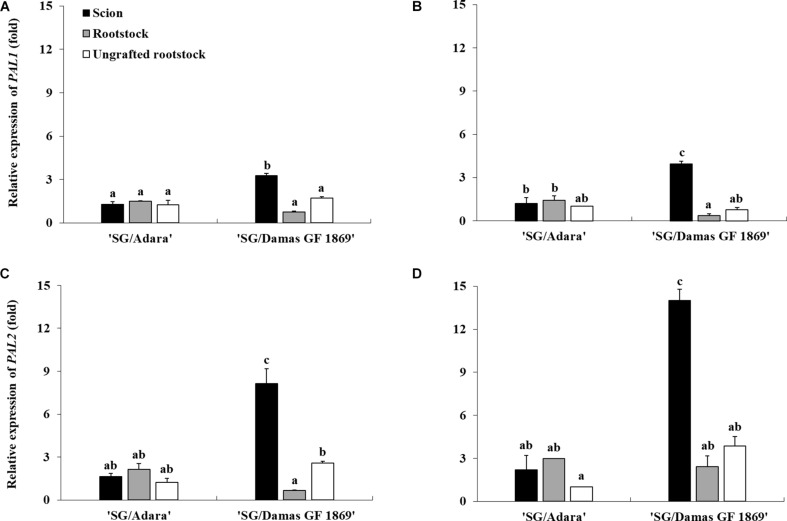
Differential expression of *PAL1* and *PAL2* genes in the bark from the scion and rootstock of the compatible (“SG/Adara”) and incompatible (“SG/Damas GF 1869”) graft-combinations, and ungrafted rootstocks [**(A,C)** October 2017; **(B,D)** June 2018] 3 years after grafting. Vertical bars represent the mean ± SE (*n* = 3 replicates). For each graph, ^*abc*^Mean values are significantly different at the *P* ≤ 0.05 level according to Duncan’s Multiple Range Test.

To identify putative regulation of the PAL activity, a gene expression analysis of two *PAL* isoforms (*PAL1* and *PAL2*) was performed in this study. The NormFinder algorithm, used to identify the stability of the four reference genes expression assessed, indicated that the most stable gene under the experimental conditions was *RPII* (Supplementary File 1). Thus, the gene expression analysis, using the *RPII* as reference gene and the ungrafted rootstock “Adara” as control, showed higher expression of both *PAL* genes in the incompatible graft-combination. Moreover, *PAL* genes expression was significantly higher above than below the graft-union only in the incompatible graft-combination. The incompatible rootstock (“Damas GF 1869”) induced approximately three-fold more expressed the *PAL1* gene in the scion than the compatible one in both sampling periods (Figures 4A,B). A more acute trend was obtained studying the *PAL2* expression, which showed five-fold (in October) and six-fold (in June) higher expression in the scion when grafted on “Damas GF 1869” than on the compatible rootstock (Figures 4C,D). For the *PAL1* expression, similar values were found in both sampling periods. However, for the *PAL2* gene, which was more induced by the graft-incompatibility, approximately two-fold higher expression was observed in the most active vegetative period (June) than at leaf fall period (October). Regarding the compatible graft-combination, no significant differences of *PAL* genes expression were found between scion and rootstock in both sampling periods. Likewise, there were no significant differences between the ungrafted rootstocks (“Adara”, “Damas GF 1869”), showing similar values as the compatible graft-combination (“S/Adara”).

### Phenotypic Correlations Between Biochemical and Molecular Parameters and Principal Components Analysis (PCA)

**FIGURE 5:**
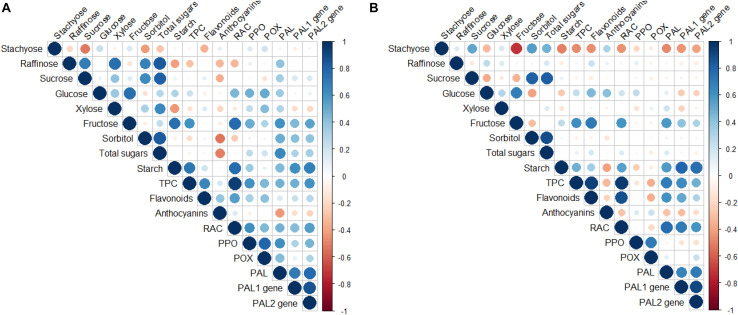
Pearson’s correlation coefficient for the traits studied in the compatible (“SG/Adara”) and incompatible (“SG/Damas GF 1869”) graft-combinations, and ungrafted rootstocks at the leaf fall **(A)** and vegetative **(B)** periods, 3 years after grafting. PAL, Phenylalanine ammonia-lyase enzyme; PPO, Polyphenol oxidase enzyme; POX, peroxidase enzyme; RAC, Relative antioxidant capacity; TPC, Total phenolics content.

Significant correlations were found between the different biochemical and molecular parameters measured at both periods (Figures 5A,B, Supplementary Files 2,3).

The PAL enzymatic activity was positively correlated with the TPC (*r* = 0.698; *P* ≤ 0.01), flavonoids (*r* = 0.60; *P* ≤ 0.01), and RAC (*r* = 0.762; *P* ≤ 0.01) at the vegetative period. These correlations suggest that the change in the PAL activity might be a principal factor in the accumulation of phenolic compounds in the scion of the incompatible graft-combination. Moreover, a positive correlation was found between the PPO enzymatic activity and TPC (*r* = 0.606; *P* ≤ 0.01), and RAC (*r* = 0.611; *P* ≤ 0.01) at leaf fall (Figure 5A and Supplementary File 2).

Some non-structural carbohydrates were also positively correlated with the phenolic compounds, antioxidant capacity, PAL activity and *PAL* genes expression. In fact, a significant and positive correlation was found between the expression of both *PAL* genes and the starch content, in both sampling periods. In addition, the fructose sugar was also positively correlated with starch content (*r* = 0.75; *P* ≤ 0.01), PPO (*r* = 0.491; *P* ≤ 0.05), and PAL activities (*r* = 0.604; *P* ≤ 0.01), and *PAL2* gene expression (*r* = 0.538; *P* ≤ 0.05) at the leaf fall period. Fructose also showed positive correlations with TPC (*r* = 0.614; *P* ≤ 0.01), flavonoids (*r* = 0.715; *P* ≤ 0.01), and RAC (*r* = 0.559; *P* ≤ 0.05) at the vegetative period (Figure 5B and Supplementary File 3). Similarly, a positive correlation was found between both *PAL* genes expression and PAL enzymatic activity (*r* = 0.70; *P* ≤ 0.01).

**FIGURE 6:**
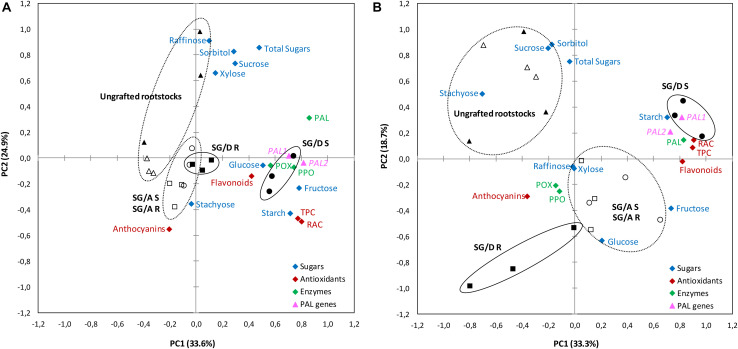
Principal component analysis (PCA) for biochemical and molecular traits evaluated on “Summergrand” (SG) cultivar budded on “Adara” and “Damas GF 1869” rootstocks at the leaf fall **(A)** and vegetative **(B)** periods, 3 years after grafting. “SG/D” S (●), “SG/Damas GF 1869” Scion; “SG/D” R (■), “SG/Damas GF 1869” Rootstock; “SG/A” S (○), “SG/Adara” Scion; “SG/A” R (□), “SG/Adara” Rootstock; Ungrafted “Damas GF 1869” (▲); Ungrafted “Adara” (Δ). PAL, Phenylalanine ammonia-lyase enzyme; PPO, Polyphenol oxidase enzyme; POX, peroxidase enzyme; RAC, Relative antioxidant capacity; TPC, Total phenolics content; TSS, Total soluble sugars.

The principal components analysis (PCA) showed that more than 50% of the observed variance could be explained by the first two components in both sampling periods (Figures 6A,B). Interestingly, the PC1 loadings showed that the incompatible scion (found in the positive side), exhibited higher values of TPC, flavonoids, RAC, starch content, PAL activity and *PAL* genes expression. In turn, the rootstock part (found at the opposite side of PC1 and PC2 at the vegetative period), indicated lower values for carbohydrates, antioxidants compounds and enzymes, showing the reciprocal effect of scion incompatibility. Moreover, whereas for the incompatible graft-combination (“SG/Damas GF 1869”), scion and rootstock were grouped separately, in the case of the compatible graft-combination (“S/Adara”), both scion and rootstock were found in the same group. The ungrafted rootstocks seem to be less explained by the PC1 loadings compared to the PC2 ones.

## Discussion

The establishment of correlations between the biochemical and molecular parameters measured in the scion and rootstock of the two studied graft combinations may reduce the number of characters for screening genotypes or progenies for graft compatibility studies, as reported by Irisarri et al. (2019). In addition, it will help to understand the mechanisms underlying scion-rootstock interactions aiming to optimize time and labor required for evaluation of a high number of grafts per individuals before releasing new rootstocks or scions to the market.

The knowledge of the biological processes involved in grafting, including wound healing, tissue fusion, and vascular reconnection, remains limited (Pina et al., 2017). In *Prunus* species, graft incompatibility responses can occur from months to years after grafting (Pina et al., 2017; Reig et al., 2019). Although most studies focused on “localized” incompatibility at early stages of development, little is known about the biochemical and molecular processes associated to the “translocated” incompatibility. In the present study, typical symptoms of the “translocated” incompatibility were detected in “SG/Damas GF 1869” trees with lower leaf SPAD index values unlike the “SG/Adara” combination, which exhibited good graft-compatibility. Similar results were found by Moreno et al. (1993) in nectarine cultivars budded on myrobalan × myrobalan clones (*P. cerasifera*), Zarrouk et al. (2006) in nectarines on different plum based rootstocks, as well as Neves et al. (2017) in peach cultivars on “Myrobalan 29C” and “Marianna 2624” plum rootstocks. Lower SPAD values could be associated with the blockage of carbohydrate assimilation and nitrogen starvation in the aerial parts (Moreno et al., 1994). In fact, another effect of grafting observed in the present study is the **alteration of the carbohydrate balance between rootstock and scion**, due to the effect of graft-incompatibility but also as consequence of grafting interaction. Changes in sugar and starch accumulation were observed in different species during early stages of graft union formation and development, and as indicator of graft incompatibility (Moing et al., 1990; Andrews and Serrano, 1993; Zarrouk et al., 2010). Sorbitol and sucrose were the individual soluble sugars identified with higher concentrations in the bark stem of scion and both rootstocks, followed by fructose and glucose, and stachyose, raffinose, and xylose, as previously reported in plum phloem tissues (Moing et al., 1987), roots (Moreno et al., 1993), and leaves (Moing et al., 1990). The accumulation of starch and sorbitol in the scion of the incompatible graft-combination (“SG/Damas GF 1869”) was previously found in other peach/plum graft-combinations exhibiting graft-incompatibility (Breen and Muraoka, 1975; Moing et al., 1987). The enhanced concentration of some non-structural carbohydrates at the scion part may be probably a result of disturbances in starch hydrolysis (Drzewiecka et al., 2012) or because of problems in cambium division at the graft-union preventing vascular tissue development and successful connection between the graft-components (Zarrouk et al., 2010). In addition, the **accumulation of assimilates in the aerial parts** can result in changes in the rate of photosynthesis and disturbances in the sink/source balance within a plant (Borowiak et al., 2015). Our results confirmed that the “translocated” graft-incompatibility may affect the non-structural carbohydrates transport between the graft-components and induce a blockage of starch and sorbitol above the graft-union. This blockage confirmed the disturbance in the phloem transport of starch and soluble sugars from the aerial part to the rootstock.

Similarly, the graft-incompatible rootstock induced higher TPC, flavonoids and RAC activity as well as higher enzymatic activities of PAL, PPO and POX in the scion. Thus, the enhancement of metabolic activities involved in **accumulation of phenolic compounds, ROS defense and increased biochemical activity** were observed in response to the incompatibility reaction. Nevertheless, the process of grafting could be also inducing an oxidative stress, even in the case of a good scion-rootstock graft compatibility, and thus affect metabolite profile in a lesser extent. In fact, ungrafted rootstocks seem to be less influenced by those metabolic activities when compared to compatible grafts, as it was expected (Pina et al., 2017). Phenolic compounds are secondary metabolites implicated in processes of division, development, differentiation into new tissues, lignification and in generating a biochemical response to biotic and abiotic stresses (Lattanzio, 2013). During the graft-union formation, an intense production of these compounds occurs. As one of the main components of plant cell wall, lignin metabolism has a great importance in plant growth and development, enhancing plant cell wall rigidity and reinforcing the plant vascular system to transport water and minerals (Liu et al., 2018). Thus, the lignin inhibition results in metabolic and growth dysfunctions and weak graft-union in graft-combinations showing “localized” graft-incompatibility (Reig et al., 2018b, 2019). On the other hand, phenolic compounds can affect the auxin plant hormone (IAA), in which monohydroxy B-ring flavonoids act as a cofactor of POX enzyme resulting in the IAA degradation (Feucht and Kahn, 1973). This growth hormone plays a major role in the differentiation of vascular tissues at the graft-union, and promotes the xylem and phloem formation (Baron et al., 2019). Thus, an inhibition of auxin transport can lead to a failure in the vascular connections establishment between the rootstock and scion in some incompatible grafts (Aloni et al., 2010). Although the accumulation of some flavonoids has been already reported in various incompatible scion/rootstock combinations of fruit trees (Hudina et al., 2014; Irisarri et al., 2015; Pina et al., 2017), to date there have been very few reports on the accumulation of phenylpropanoid and antioxidant enzyme activities related to the “translocated” incompatibility. In the present work, higher PAL activity was only observed in the scion of the incompatible graft-combination, as previously reported by Pereira et al. (2014). Similarly, Musacchi et al. (2002) and Zarrouk et al. (2010) also showed higher POX activity in scions of nectarine/plum and pear/quince incompatible graft-combinations.

On the other hand, oxidative stress has been implicated in the graft-incompatibility response of herbaceous and woody plants (Aloni et al., 2008; Nocito et al., 2010; Irisarri et al., 2015). The excessive accumulation of reactive oxygen species (ROS), that can be a consequence of graft-incompatibility stress, lead to the disruption of the normal physiological and cellular functions resulting in cell walls damage and oxidative stress (Pandey et al., 2017). As a response, the plant trigger sits antioxidative defense systems composed of antioxidant enzymes such as the POX enzyme, to get rid of these ROS and promote wound healing (Xu et al., 2015). In our study, the abnormal increase of POX activity observed in the incompatible graft-combination can be also explained by an antioxidant defense reaction triggered by the tree due to a different oxidative stress. Moreover, it has been shown that POX activity is strictly linked to the tissue content of flavanols, a subgroup of flavonoids that are involved in the non-enzymatic antioxidative defense system of plants (Musacchi et al., 2002; Racchi, 2013). This might also explain the flavonoids increase in the incompatible graft-combination in our study. In addition, POX enzyme is also involved in the oxidation of auxin, inducing depressed levels of indole-3-acetic acid (IAA) and affecting plant growth and development as above mentioned (Pandey et al., 2017). Likewise, PPO enzymes play a paramount role as ROS inducers and can trigger cell death during a severe oxidative stress (Musacchi et al., 2002). Thus, indirectly, high PPO activity may adversely affect cell differentiation and proliferation impairing a good connection between scion and rootstock (Porfírio et al., 2016). A positive correlation was found between the PPO enzymatic activity and TPC and RAC at leaf fall period. These correlations can be explained by the role of the carbohydrates in the regulation of the phenolic compounds biosynthesis. The increase in fructose content in the incompatible scion might enhance the erythose-4-phosphate production that constitute, together with the phosphoenol pyruvate, a substrate for lignin and phenolic compounds through the shikimate pathway as reported by Ibrahim and Jaafar (2011) in non-grafted plants. The secondary metabolism (phenolic compounds) is linked to primary metabolism (soluble sugars and starch) by the rates at which substrates are diverted from primary pathways and funneled into the secondary biosynthetic routes. Thus, abiotic and biotic stresses affecting growth, photosynthesis and other parts of primary metabolism will also affect secondary metabolism (Ibrahim and Jaafar, 2011). The activation of repair mechanisms during the stress processes associated to grafting could need to supply carbon skeletons, synthesize new molecules or increase antioxidative enzyme activity. Many of these processes can be supported by photosynthetic activity (Aloni et al., 2008; Calatayud et al., 2013; Penella et al., 2014; Pina et al., 2017).

In addition to the accumulation of metabolites, **differences in *PAL* gene expression** were previously reported in apricot cultivars grafted on plum rootstocks (Pina and Errea, 2008; Irisarri et al., 2016) and in peach grafted on Japanese apricot rootstocks 2 years after grafting (Pereira et al., 2014) showing “localized” incompatibility. Pereira et al. (2014), studying the same *PAL* peach isoforms, found a higher *PAL1* expression than *PAL2*. In contrast, in our study, a higher over-expression of *PAL2* was observed in the incompatible graft-combination compared with *PAL1*. This may be due to differences of plant material and the different type of incompatibility considered. Under stress, the transcription of messenger RNA that codes for PAL is triggered, resulting in an increase of the PAL synthesis in the plant and stimulating the synthesis of phenolic compounds (Lillo et al., 2008). Furthermore, the positive correlation found between both *PAL* genes expression and PAL enzymatic activity was previously reported in the “localized” type of incompatibility (Pereira et al., 2014). The differential expression of *PAL* genes might be associated with the higher PAL enzymatic activity, observed in the scion grafted on the incompatible rootstock. Similarly, *PAL* genes showed positive and significant correlations with the total phenolics content (TPC), flavonoids and the antioxidant capacity (RAC). The relationship between PAL activity, phenolics accumulation, and RAC was reported in peach/Japanese apricot graft-combinations in response to “localized” incompatibility stress (Pereira et al., 2018).

## Conclusion

In the present study, it was possible to reveal the good correlation between the biochemical and molecular parameters, and “translocated” incompatibility across two peach/plum graft-combinations. To date, no previous studies have been reported describing the *PAL* genes expression in response to “translocated” peach/plum graft-incompatibility. Moreover, the level of transcription of the *PAL* genes can differentiate between compatible and incompatible graft-combinations. It is suggested that the over-expression of these genes constitute a good indicator of the onset of the “translocated” graft-incompatibility. Moreover, the differential expression of both *PAL* genes was associated with the higher PAL enzymatic activity observed in the scion grafted on the incompatible rootstock. This can provide key information for the genes related to the incompatibility between the scion and rootstock. Together with the molecular effects, the high activities of three enzymes (PAL, PPO, and POX) resulted in an enhanced production of phenolic compounds (total phenolic content and flavonoids) as well as the antioxidant activity in the scion of the incompatible graft-combination. Further studies in the identification and transcription of genes involved in other metabolic pathways related to the PPO and POX enzymes could be interesting in the investigation of this type of graft-incompatibility in *Prunus.* The identification of mRNA and small RNA graft-transmissible signals that may play a pivotal role in grafting physiology could be a step further to this investigation.

## Data Availability Statement

The raw data supporting the conclusions of this article will be made available by the authors, without undue reservation.

## Author Contributions

MM and CF conceived and designed the experiments, contributed with reagents, materials, and analysis tools. RA, CF, RG, and MM performed the experiments and analyzed the data. All authors discussed the results, contributed to the manuscript preparation, and read and approved the final version.

## Conflict of Interest

The authors declare that the research was conducted in the absence of any commercial or financial relationships that could be construed as a potential conflict of interest.

## Abbreviations

*ACT11*: Actin
Cq: quantification cycles
non-structural carbohydrates (NSC), PAL: phenylalanine ammonia-lyase
POX: peroxidase
PPO: polyphenol oxidase
RAC: relative antioxidant capacity
*RP II*: RNA polymerase II
SPAD: soil plant analysis diagnostic
TCSA: trunk cross section area
*TEF2*: translation elongation factor 2.
