# Supplementary material for: Biochemical Characterization and Differential Expression of PAL Genes Associated With “Translocated” Peach/Plum Graft-Incompatibility
Source: Front Plant Sci. 2021 Feb 19;12:622578. doi: 10.3389/fpls.2021.622578 (PMC7933046; doi:10.3389/fpls.2021.622578)
Supplement: Supplementary file 1 [file Table_1.docx]

**Supplementary File 1.**

Stability values of the four candidate genes (*Actin*, *EST*, *TEF2* and *RPII*) performed by NormFinder algorithm adapted for Microsoft Excel software.

|  | Stability value | |
| --- | --- | --- |
|  | Leaf fall | Vegetative period |
| *Actin* | 0.127 | 0.194 |
| *EST* | 0.108 | 0.230 |
| *TEF2* | 0.098 | 0.189 |
| *RP II* | 0.034 | 0.042 |
