# Supplementary material for: Biochemical Characterization and Differential Expression of PAL Genes Associated With “Translocated” Peach/Plum Graft-Incompatibility
Source: Front Plant Sci. 2021 Feb 19;12:622578. doi: 10.3389/fpls.2021.622578 (PMC7933046; doi:10.3389/fpls.2021.622578)
Supplement: Supplementary file 2 [file Table_2.docx]

**Supplementary File 2.**

Pearson’s correlation coefficient for the traits studied in the compatible (“SG/Adara”) and incompatible (“SG/Damas GF 1869”) graft-combinations, and ungrafted rootstocks at the leaf fall period.

| Trait | Sucrose | Xylose | Fructose | Sorbitol | TSS | Starch | TPC | Flavonoids | Anthocyanins | RAC | PPO activity | POX activity | PAL activity | *PAL 1* expression | *PAL 2* expression |
| --- | --- | --- | --- | --- | --- | --- | --- | --- | --- | --- | --- | --- | --- | --- | --- |
| Stachyose  Raffinose  Sucrose  Glucose  Xylose  Fructose  Sorbitol  TSS  Starch  TPC  Flavonoids  RAC  PPO activity  PAL activity  *PAL1* expression | -0.523^*^  0.693^**^  - | ns  0.759^**^  ns  ns  - | ns  ns  ns  0.759^**^  ns  - | ns  0.676^**^  0.638^**^  ns  ns  ns  - | ns  0.834^**^  0.818^**^  ns  0.645^**^  ns  0.831^**^  - | ns  ns  ns  ns  ns  0.750^**^  ns  ns  - | ns  ns  ns  ns  ns  0.605^**^  ns  ns  0.730^**^  - | ns  ns  ns  ns  ns  ns  ns  ns  ns  0.686^**^  - | ns  ns  ns  ns  ns  ns  -0.538^*^  -0.509^*^  ns  ns  ns | ns  ns  ns  ns  ns  0.773^**^  ns  ns  0.765^**^  0.935^**^  0.600^**^  - | ns  ns  ns  0.482^*^  ns  0.491^*^  ns  ns  ns  0.606^**^  ns  0.611^**^  - | ns  ns  ns  0.506^*^  ns  ns  ns  ns  ns  ns  ns  ns  0.790^**^ | ns  ns  ns  ns  ns  0.604^**^  0.498^*^  0.645^*^  ns  0.479^*^  ns  0.480^*^  0.613^*^  - | ns  ns  ns  ns  ns  ns  ns  ns  0.619^**^  0.483^*^  ns  ns  ns  0.701^**^  - | ns  ns  ns  ns  ns  0.538^*^  ns  ns  0.685^**^  0.604^**^  ns  0.551^*^  ns  0.777^**^  0.863^**^ |

* Correlation is significant at the 0.05 level (bilateral).

** Correlation is significant at the 0.01 level (bilateral).

ns: non significant; TSS: Total Soluble Sugars; TPC: Total Phenolics Content.
