# Supplementary material for: Biochemical Characterization and Differential Expression of PAL Genes Associated With “Translocated” Peach/Plum Graft-Incompatibility
Source: Front Plant Sci. 2021 Feb 19;12:622578. doi: 10.3389/fpls.2021.622578 (PMC7933046; doi:10.3389/fpls.2021.622578)
Supplement: Supplementary file 3 [file Table_3.docx]

**Supplementary File 3.**

Pearson’s correlation coefficient for the traits studied in the compatible (“SG/Adara”) and incompatible (“SG/Damas GF 1869”) graft-combinations, and ungrafted rootstocks at the vegetative period.

| Trait | Sucrose | Fructose | Sorbitol | TSS | Starch | TPC | Flavonoids | RAC | POX  activity | PAL activity | *PAL 1* expression | *PAL 2* expression |
| --- | --- | --- | --- | --- | --- | --- | --- | --- | --- | --- | --- | --- |
| Stachyose  Sucrose  Glucose  Fructose  Sorbitol  Starch  TPC  Flavonoids  RAC  PPO activity  PAL activity  *PAL1* expression | - 0.524^*^  - | - 0.721^**^  ns  0.690^**^  - | 0.552^*^  0.819^**^  ns  ns  - | 0.475^*^  0.823^**^  ns  ns  0.888^**^ | - 0.500^*^  ns  ns  ns  ns  - | - 0.474^*^  ns  ns  0.614^**^  ns  0.488^*^  - | ns  ns  ns  0.715^**^  ns  ns  0.935^**^  - | ns  ns  ns  0.599^**^  ns  0.544^*^  0.958^**^  0.871^**^  - | ns  ns  ns  ns  ns  ns  ns  ns  ns  0.700^**^ | ns  ns  ns  ns  ns  0.562^*^  0.698^**^  0.600^**^  0.762^**^  ns  - | ns  ns  ns  ns  ns  0.792^**^  0.633^**^  0.512^*^  0.706^**^  ns  0.666^**^  - | ns  ns  ns  ns  ns  0.757^**^  0.486^*^  ns  0.597^*^  ns  0.702^**^  0.907^**^ |

* Correlation is significant at the 0.05 level (bilateral).

** Correlation is significant at the 0.01 level (bilateral).

ns: non significant; TSS: Total Soluble Sugars; TPC: Total Phenolics Content
